# Driver gene alterations in NSCLC patients in southern China and their correlation with clinicopathologic characteristics

**DOI:** 10.3389/fgene.2024.1455502

**Published:** 2024-09-19

**Authors:** Lingna Deng, Jinbang Li, Zhanlong Qiu, Yanfen Wang

**Affiliations:** Department of Pathology, The Affiliated Qingyuan Hospital (Qingyuan People’s Hospital), Guangzhou Medical University, Qingyuan, China

**Keywords:** EGFR, genomic alterations, non-small cell lung cancer, targeted therapy, KRAS

## Abstract

**Introduction:**

In this study, we aimed to explore the relationship between clinicopathological features and driver gene changes in Chinese NSCLC patients.

**Methods:**

Amplification refractory mutation system PCR was used to detect the aberrations of 10 driver oncogenes in 851 Chinese NSCLC patients, and their correlation with clinicopathological characteristics was also analyzed. Moreover, three models of logistic regression were used to analyze the association between histopathology and *EGFR* or *KRAS* mutations.

**Results:**

The top two most frequently aberrant target oncogenes were *EGFR* (48.06%) and *KRAS* (9.51%). These were followed by *ALK* (5.41%), *HER2* (2.35%), *MET* (2.23%), *RET* (2.11%), *ROS1* (1.88%), *BRAF* (0.47%), *NRAS* (0.24%), and *PIK3CA* (0.12%). Additionally, 11 (1.29%) patients had synchronous gene alterations in two genes. The main *EGFR* mutations were exon 21 L858R and exon 19-Del, which accounted for 45.97% and 42.79% of all *EGFR* mutations, respectively. Logistic regression analysis showed that the frequency of *EGFR* mutations was positively correlated with women, non-smokers, lung adenocarcinoma, and invasive non-mucinous adenocarcinoma (IA), and negatively correlated with solid nodule, micro-invasive adenocarcinoma, and solid-predominant adenocarcinoma. *KRAS* mutations were positively associated with men and longer tumor long diameters and negatively correlated with lung adenocarcinoma (*P < 0.05* for all).

**Conclusion:**

Our findings suggest that the *EGFR* mutation frequency was higher in women, non-smokers, lung adenocarcinoma, and the IA subtype in lung adenocarcinoma patients, while the *KRAS* mutation rate was higher in men and patients with longer tumor long diameter and lower in lung adenocarcinoma patients.

## 1 Introduction

Lung cancer is one of the common malignant tumors that threaten human life and health, and the most recent global cancer statistics show that lung cancer is the leading cause of cancer death (Lancet, 2024). In recent years, with the rapid development of molecular detection, more and more molecular drivers have been detected as therapeutic targets for non-small cell lung cancer (NSCLC) (Yang et al., 2022), including alterations of anaplastic lymphoma kinase (*ALK*), ROS proto-oncogene 1 (*ROS1*), rearranged during transfection (*RET*), epidermal growth factor receptor (*EGFR*), v-Ki-ras2 Kirsten rat sarcoma viral oncogene homolog (*KRAS*), human epidermal growth factor receptor 2 (*HER2*), B-raf proto-oncogene (*BRAF*), and mesenchymal-epithelial transition factor (*MET*), among which the most common is *EGFR* mutations (Di Maio, 2023). As new molecular-targeted therapies have made substantial progress in NSCLC, the survival rate of NSCLC patients with specific genotypic alterations has been significantly improved (Wu et al., 2024; Planchard et al., 2023). The latest version of NCCN Clinical Practice Guidelines in Oncology recommends that when testing for NSCLC, in addition to all of the classical actionable molecular markers (e.g., *EGFR*, *ALK*, *BRAF*, *KRAS*, *MET*ex14 skipping, *NTRK1/2*, *RET*, *ROS1*), emerging molecular markers, such as high-level *MET* amplification, and *ERBB2* mutation should also be detected (Ettinger et al., 2022).

EGFR tyrosine kinase inhibitors (EGFR-TKIs) can prolong survival and improve the quality of life of patients with NSCLC with *EGFR* mutations (Ramalingam et al., 2020). At the same time, the continuous activation mutation of *KRAS* may affect the therapeutic effect of EGFR-TKIs in patients with NSCLC. In addition, mutations in exon 20 of *EGFR*, such as T790M, may mediate resistance to EGFR-TKIs (Arbour and Riely, 2019; Tan et al., 2024). A comprehensive analysis of pathological types and tumor-specific molecular abnormalities in patients with NSCLC can help patients maximize treatment benefits while reducing treatment-related risks (Eberhardt et al., 2015). Therefore, using accurate and low-cost gene detection methods to identify the alterations of targeted driver genes in NSCLC patients and explore the correlation between them and clinicopathological features is of great significance for the individualized treatment of NSCLC patients. Some studies have suggested that lung adenocarcinoma, women, and non-smokers were positively associated with *EGFR* mutations (Li et al., 2020; Wang et al., 2020). However, other studies found no significant association between smoking status and gender with *EGFR* mutations (Ess et al., 2017). Many studies have reported that age was not associated with *EGFR* mutations (Gao et al., 2020; Zhao et al., 2018; Yang et al., 2014), while some investigators found *EGFR* mutations were more frequent in younger or older patients with NSCLC (Vallee et al., 2013; Wu et al., 2019). Thus, the role of clinicopathological characteristics, including gender, age, histology types, smoking status, etc., as predictors for the alteration of NSCLC driver genes remains controversial.

In this retrospective study, participants were recruited to undergo the detection of 10 target oncogene aberrations, including *EGFR*, *KRAS*, *MET*, *ALK*, *ROS1*, *HER2*, *RET*, *BRAF*, *NRAS*, and *PIK3CA*. The chi-squared test, Fisher exact test, and logistic regression analysis were used to analyze the relationship between 10 driver gene alterations and the clinicopathological characteristics of NSCLC patients.

## 2 Patients and methods

### 2.1 Patients

In this study, information from 851 patients with NSCLC seen between October 2019 and September 2023 in the Sixth Affiliated Hospital of Guangzhou Medical University was collected. Patients who met the following criteria were included:

(1) patients with histological or pathological diagnosis of NSCLC;
(2) complete clinical medical records.

The exclusion criterion was patients with concomitant malignant disease of other systems. This study was conducted in accordance with the Declaration of Helsinki and approved by the Ethics Committee of the Sixth Affiliated Hospital of Guangzhou Medical University. Due to the retrospective nature of the study, the Ethics Committee waived the need for patient informed consent.

### 2.2 Clinicopathologic characteristics

Clinical data were obtained from patients’ electronic medical record database, including sex, age, smoking history, drinking status, family history, serum markers of lung tumors, chest computed tomography (CT) features, and TNM stage. Serum tumor markers included carcinoma embryonic antigen (CEA), cytokeratin 19 fragment antigen21-1 (CYFRA21-1), neuron-specific enolase (NSE), pro-gastrin-releasing peptide (ProGRP), and squamous-cell carcinoma antigen (SCCA). Abnormal serum markers were defined as elevated levels of at least one of the above serum markers. TNMs were determined according to the eighth edition of the American Joint Committee on Cancer TNM staging system for lung cancer (Rami-Porta et al., 2015). Histologic subtypes of NSCLCs were classified according to the 5th edition of the World Health Organization Classification of Thoracic Tumours (2021).

### 2.3 DNA/RNA extraction and driver gene detection

We collected three types of specimens, including biopsy (504), surgical specimen (328), and malignant exudate (19). All specimens were fixed with a 10% neutral formalin solution, dehydrated, paraffin-embedded, sliced, and stained by HE. Pathologists with more than 5 years of diagnostic experience evaluated the proportion of tumor cells in the tissues for gene change detection. Tumor specimens with more than 30% tumor cells were selected, and 5–8 wax rolls of 5 μm thickness were cut and placed in an enzyme-free EP tube for DNA and RNA extraction in strict accordance with the FFEP DNA/RNA extraction kit instructions (Amoy Diagnostics Co., Ltd., Xiamen, China). In strict accordance with the kit instructions (Amoy Diagnostics Co., Ltd., Xiamen, China), we used the amplification refractory mutation system PCR (ARMs-PCR) method in the standard PCR laboratory to detect the target oncogene aberrations, including *EGFR*, *KRAS*, *MET*, *ALK*, *ROS1*, *HER2*, *RET*, *BRAF*, *NRAS*, and *PIK3CA*. (Table 1).

**TABLE 1:** Types of fusions or mutations of 10 targeted driver genes.

Molecular marker	Types of fusions or mutations
*ALK*	EML4 exon 13; ALK exon 20, EML4 exon 6 ins33; ALK exon 20, EML4 exon 20; ALK exon 20, EML4 exon 18; ALK exon 20, EML4 exon 2; ALK exon 20, EML4 exon 17; ins68 ALK exon 20*, EML4 exon 2; ins117 ALK exon 20*, EML4 exon 13; ins69 ALK exon 20*, EML4 exon 6; ALK exon 20*, EML4 exon 6; ALK exon 19*, EML4 exon 6; ins18 ALK exon 20*, EML4 exon 20; ins18 ALK exon 20*, EML4 exon 17del58; ins39 ALK exon 20*, EML4 exon 17ins65; ALK exon 20*, EML4 exon 17; ins30 ALK exon 20*, EML4 exon 17 ins61; ins34 ALK exon 20*, EML4 exon 3; ins53 ALK exon 20*, KIF5B exon 24; ALK exon 20*, KIF5B exon 17; ALK exon 20*, KLC1 exon 9; ALK exon 20*, TFG exon 4; ALK exon 20*
*RET*	CCDC6 exon 1; RET exon 12*, NCOA4 exon 6; RET exon 12*, KIF5B exon 15; RET exon 12*, KIF5B exon 16; RET exon 12*, KIF5B exon 23; RET exon 12*, KIF5B exon 22; RET exon 12*
*ROS1*	SLC34A2 exon 4; ROS1 exon 32, SLC34A2 exon 14 del; ROS1 exon 32, CD74 exon 6; ROS1 exon 32, SDC4 exon 2; ROS1 exon 32, SDC4 exon 4; ROS1 exon 32, SLC34A2 exon 4; ROS1 exon 34, SLC34A2 exon 14 del; ROS1 exon 34, CD74 exon 6; ROS1 exon 34, SDC4 exon 4; ROS1 exon 34, EZR exon 10; ROS1 exon 34 TPM3 exon 8; ROS1 exon 35, LRIG3 exon 16; ROS1 exon 35, GOPC exon 8; ROS1 exon 35
*EGFR*	G719A, G719C, G719S*; 19-Del; T790M; 20-ins*; S768I; L858R; L861Q
*KRAS*	G12C, G12R, G12V, G12A, G13C; G12S, G12D
*NRAS*	Q61R*, Q61K*, Q61L*, Q61H*
*BRAF*	V600E, V600K*, V600E2*, V600R*, V600D1*, V600D2*
*HER2*	A775_G776insYVMA*, M774_A775insAYVM*; G776>VC*, G776>LC*, P780_Y781insGSP*
*PIK3CA*	H1047R*, E545K*
*MET*	Exon 14 skipping mutation

### 2.4 Statistical analysis

All statistical analyses were performed using SPSS 27.0 software (SPSS, Armonk, NY, United States). A Student’s t-test was used to examine the association of tumor long diameter with the 10 target genes. The chi-squared test or Fisher exact test was used to explore the association of other clinicopathological features with 10 targeted gene alterations. A logistic regression analysis was performed to assess possible confounding factors between driver gene alterations and histology types, subtypes of adenocarcinoma subtypes, and subtypes of invasive adenocarcinoma (Model 1). Preliminary adjustment covariates included gender, age, and smoking history (Model 2), while full adjustment covariates included gender, age, smoking history, drinking status, serum tumor markers, pulmonary nodule types, tumor long diameter, and TNM stage (Model 3). All tests were two-sided, and a *P*-value < 0.05 was considered statistically significant.

## 3 Results

### 3.1 Demographics and histopathological characteristics of the NSCLC patients

Table 2 summarizes the demographics, clinical characteristics, biochemical parameters, and imaging findings in patients with NSCLC. Originally, 857 patients were included in this study, but six patients were excluded due to concurrent systemic malignancies, and 851 patients were analyzed. The histopathologic diagnosis of the NSCLC cases included in this study was confirmed by one or more thoracic pathologists. Overall, in 851 cases of non-small-cell lung cancer, 492 (57.81%) patients were men, 360 (42.30%) patients were younger than 60 years, 345 (40.54%) patients had a history of smoking, and 95 (11.16%) patients had a history of drinking. Of the complete cohort, 668 (78.50%) patients had abnormal serum tumor markers; 337 (39.60%) patients were in the early stage (including TNM stages 0, I, and II) of lung cancer, and the remaining 514 (60.40%) patients were in the advanced stage (including TNM stages III and IV) of lung cancer.

**TABLE 2:** Demographics, clinical characteristics, biochemical parameters, and imaging findings in patients with NSCLC.

Characteristic	No. of patients (%)
Sex, male	492 (57.81)
Age, ≤ 60 years	360 (42.30)
Smoking habit	345 (40.54)
Drinking status	95 (11.16)
Abnormal serum tumor markers	668 (78.50)
Tumor location	
Upper right lung	273 (32.08)
Middle right lung	123 (14.45)
Lower right lung	145 (17.04)
Upper left lung	210 (24.68)
Lower left lung	100 (11.75)
Tumor long diameter (cm)	
<1	55 (6.46)
1–3	395 (46.42)
>3	401 (47.12)
Pulmonary nodule types	
Solid nodule	727 (85.43)
Part-solid nodule	59 (6.93)
Ground-glass nodule	65 (7.64)
TNM stage	
0	3 (0.35)
I	299 (35.13)
II	35 (4.11)
III	125 (14.69)
IV	389 (45.71)

**FIGURE 1:**
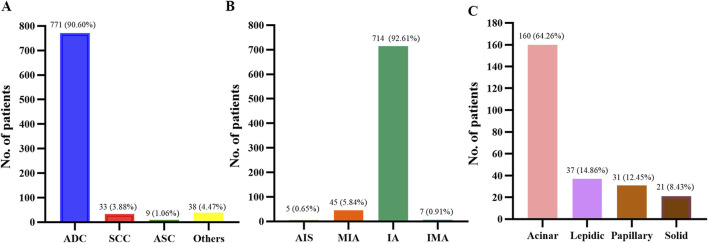
Histopathology of patients. **(A)** Histology types of 851 patients with NSCLC. (**B)** Histological subtypes of 771 patients with lung adenocarcinoma. **(C)** Histological subtypes of 249 patients with lung invasive adenocarcinoma. NSCLC, non-small cell lung cancer; ADC, adenocarcinoma; SCC, squamous cell carcinoma; ASC, adenosquamous carcinoma; AIS, adenocarcinoma *in situ*; MIA, micro-invasive adenocarcinoma; IA, invasive non-mucinous adenocarcinoma; IMA, invasive mucinous adenocarcinoma.

As shown in Figure 1, among the 851 patients with NSCLC, 771 (90.60%) patients had adenocarcinoma (ADC), 33 (3.88%) patients had squamous-cell carcinoma (SCC), nine (1.06%) patients had adenosquamous carcinoma (ASC) and 38 (4.47%) patients had other subtypes of NSCLC. Of those 771 ADC patients, five (0.65%) patients had adenocarcinoma *in situ* (AIS), 45 (5.84%) patients had minimally invasive adenocarcinoma (MIA), 714 (92.61%) patients had invasive non-mucinous adenocarcinoma (IA), and seven (0.91%) patients had invasive mucinous adenocarcinoma (IMA). For patients with IA, 249 patients were classified, as only surgical specimens could be further classified. Amongst the 249 IA patients, acinar-predominant adenocarcinoma was the most common (64.26%), followed by lepidic-predominant adenocarcinoma (14.86%), 31 papillary-predominant adenocarcinoma (12.45%), and solid-predominant adenocarcinoma (8.43%).

### 3.2 Detection of 10 target gene alterations in NSCLC

Among the 851 patients with NSCLC, 627 cases (73.68%) had alterations in at least one of the 10 target genes (Supplementary Figure S1A). As shown in Supplementary Figure S1B, the top two most frequently aberrant target oncogenes were *EGFR* (48.06%) and *KRAS* (9.51%). The remaining eight target oncogenes aberrations were *ALK* (5.41%), *HER2* (2.35%), *MET* (2.23%), *RET* (2.11%), *ROS1* (1.88%), *BRAF* (0.47%), *NRAS* (0.24%), and *PIK3CA* (0.12%). Relationships between 10 target gene alterations and patient characteristics are shown in Table 3. In general, the frequency of alterations in 10 target genes was significantly higher in women, never smokers, never drinkers, patients with normal serum tumor markers, patients in the early stage (*p = 0.019*), and shorter tumor long diameters. There was a prevalence of more target gene alterations in ADC than in other histology types. Target gene alterations were more common in patients with part-solid nodules than solid nodules and in patients with ground-glass nodules than that of solid nodules. There was no significant correlation between target gene alterations and age or tumor location. Additionally, Table 4 shows that 11 patients (1.29%) had synchronous gene alterations in two genes, 10 of which were *EGFR* mutations combined with mutations or rearrangements of other nine targeted driver genes, and the remaining case was a *KRAS* G12C, G12R, G12V, G12A, G13C/*MET* exon 14 skipping mutation.

**TABLE 3:** Comparison of the mutation rate of 10 targeted genes in different clinicopathologic characteristics.

Clinicopathologic characteristic	No. of patients	Mutation	Mutation rate (%)	*P*
Sex				< 0.001*
Male	492	297	60.37	
Female	359	330	91.92	
Age				*0.664*
≤ 60	360	268	74.44	
> 60	491	359	73.12	
Smoking habit				< 0.001*
Never	506	437	86.36	
Former/current	345	190	55.07	
Drinking habit				< 0.001*
Never	756	577	76.32	
Former/current	95	50	52.63	
Serum tumor markers				0.021*
Normal	183	147	80.33	
Abnormal	668	480	71.86	
Tumor location				*0.073*
Upper right lung	273	192	70.33	
Middle right lung	123	100	81.30	
Lower right lung	145	114	78.62	
Upper left lung	210	147	70.00	
Lower left lung	100	74	74.00	
Pulmonary nodule types				< 0.001*
Solid nodule	727	513	70.56	
Part-solid nodule	59	57	96.61	
Ground-glass nodule	65	57	87.69	
Tumor long diameter (mean ± SD)				0.013*
With target gene alterations	3.39 ± 2.40			
Without target gene alterations	3.86 ± 2.47			
Histology types				< 0.001*
ADC	771	612	79.38	
SCC	33	2	6.06	
ASC	9	4	44.44	
Others	38	9	23.68	
TNM stage				0.019*
0 + I + II	337	263	78.04	
III + IV	514	364	70.82	

*indicates *P*-value < 0.05; ADC, adenocarcinoma; SCC, squamous cell carcinoma; ASC, adenosquamous carcinoma.

**TABLE 4:** Clinicopathologic features of NSCLC patients with synchronous gene alterations.

Case	Mutation site	Age/gender	Smoking	Histology type	Subtypes of ADC	Subtypes of IA
1	EGFR 19-Del/PIK3CA	54/FM	Never	ASC	—	—
2	EGFR 19-Del/PIK3CA	49/FM	Never	ADC	IA	Acinar
3	EGFR 19-Del/PIK3CA	64/M	Former	ADC	IA	Acinar
4	EGFR 19-Del/PIK3CA	74/FM	Never	ADC	IA	—
5	EGFR 19-Del/KRAS G12S and G12D	58/FM	Never	ADC	IA	—
6	EGFR 19-Del/KRAS G12C, G12R, G12V, G12A, and G13C	73/FM	Never	ADC	IA	—
7	EGFR 19-Del/RET	63/M	Never	ADC	IA	—
8	EGFR 19-Del/ALK	66/FM	Never	ADC	IA	—
9	EGFR 20-ins/MET	69/FM	Never	ADC	IA	Lepidic
10	EGFR L858R/BRAF	74/FM	Never	ADC	IA	—
11	KRAS G12C, G12R, G12V, G12A, and G13C/MET	50/FM	Never	ADC	IA	Lepidic

NSCLC, non-small cell lung cancer; ADC, adenocarcinoma; ASC, adenosquamous carcinoma; IA, invasive non-mucinous adenocarcinoma.

### 3.3 Relationship between *EGFR* mutations and patient clinicopathologic characteristics

Among 409 patients with *EGFR* mutations, the two most prevalent mutation subtypes were exon 21 L858R and exon 19-Del, which accounted for 22.09% and 20.56% of the total 10 targeted gene alterations, and 45.97% and 42.79% of total *EGFR* mutations, respectively. The other three mutation subtypes were exon 20 ins (14 patients, 3.42%), exon 18 G719X (8 patients, 1.96%), and exon 21 L861Q (6 patients, 1.47%). Moreover, 18 of 409 *EGFR* mutation patients (4.40%), all of whom were lung adenocarcinoma patients, harbored two coexisting subtypes of *EGFR*. There were patients with S768I combined with other *EGFR* mutations, the most common being G719X (6/9), followed by S768I/L858R (2/9) and S768I/19-Del (1/9). Six patients had T790M combined with other *EGFR* mutations, including five cases of T790M/19-Del mutations and one case of T790M/L858R. The remaining two cases had 19-Del/L858R mutations, and one case had a G719X/L861Q mutation (Supplementary Table S1).

Relationships between *EGFR* mutations and clinicopathologic characteristics of patients are shown in Table 5. Overall, *EGFR* mutations were more frequent in women than men (69.36% *vs.* 32.52%, *P< 0.001*), more frequent in never smokers than former/current smokers (61.26% *vs.* 28.70%, *P< 0.001*), more frequent in never drinkers than drinkers (50.40% *vs.* 29.47%, *P< 0.001*), more frequent in patients with normal serum tumor markers than abnormal serum tumor makers (54.64% *vs.* 46.26%, *P = 0.044*), and more frequent in patients with early stage than advanced stage (58.16% *vs.* 41.44%, *P< 0.001*). The frequency rate of *EGFR* mutations was lower in patients with solid nodules than in patients with ground-glass nodules (43.60% *vs.* 69.23%, *P< 0.001*) and was higher in patients with shorter tumor long diameters than in patients with longer tumor long diameters (3.15 ± 2.23 *vs.* 3.84 ± 2.56, *P< 0.001*). Furthermore, the mutation rate of *EGFR* in 771 adenocarcinoma patients (n = 407, 52.79%) was higher than that in patients with other types of NSCLC (*P < 0.001*). There was no significant relationship between age and total *EGFR* mutations.

**TABLE 5:** Relationship between *EGFR* gene mutations and clinicopathologic characteristics in patients with NSCLC.

Clinicopathologic characteristic	No. of patients						
	No. of patients	Total *EGFR* mutations	18 G719X	19 Del	20 ins	21 L858R	21 L861Q
No. of patients		409	8	175	14	188	6
Sex (*P*)		< 0.001*	*0.293*	< 0.001*	0.005*	< 0.001*	*0.088*
Male	492	160	3	72	3	69	1
Female	359	249	5	103	11	119	5
Age (*P*)		*0.998*	0.024*	*0.232*	*0.966*	*0.672*	*0.248*
≤ 60	360	173	0	81	6	77	4
> 60	491	236	8	94	8	111	2
Smoking habit (*P*)		< 0.001*	*0.484*	< 0.001*	0.044*	< 0.001*	*0.087*
Never	506	310	6	131	12	141	6
Former/current	345	99	2	44	2	47	0
Drinking habit (*P*)		< 0.001*	*0.608*	*0.076*	*0.387*	*0.067*	*0.490*
Never	756	381	8	162	14	174	6
Former/current	95	28	0	13	0	14	0
Serum tumor markers (*P*)		0.044*	*0.213*	*0.268*	0.016*	*0.605*	*0.614*
Normal	183	100	0	43	7	43	2
Abnormal	668	309	8	132	7	145	4
Pulmonary nodule types (*P*)		< 0.001*	*0.514*	0.002*	*0.852*	< 0.001*	*0.403*
Solid nodule	727	317	7	137	13	143	5
Part-solid nodule	59	47	1	22	0	20	1
Ground-glass nodule	65	45	0	16	1	25	0
Tumor long diameter (*P*)		< 0.001*	*0.857*	0.009*	*0.139*	*0.055*	*0.328*
With alteration (mean ± SD)		3.15 ± 2.23	3.36 ± 2.01	3.13 ± 2.07	2.56 ± 1.78	3.22 ± 2.35	2.55 ± 2.25
Without alteration (mean ± SD)		3.84 ± 2.56	3.52 ± 2.43	3.62 ± 2.51	3.53 ± 2.44	3.60 ± 2.45	3.52 ± 2.43
Histology types (*P*)		< 0.001*	*0.840*	< 0.001*	*0.576*	< 0.001*	*0.890*
ADC	771	407	8	174	14	187	6
SCC	33	0	0	0	0	0	0
ASC	9	0	0	0	0	0	0
Others	38	2	0	1	0	1	0
TNM stage (*P*)		< 0.001*	*0.064*	*0.182*	*0.422*	< 0.001*	*0.221*
0 + I + II	337	196	6	77	7	95	4
III + IV	514	213	2	98	7	93	2

*indicates *P*-value < 0.05; NSCLC, non-small cell lung cancer; ADC, adenocarcinoma; SCC, squamous-cell carcinoma; ASC, adenosquamous carcinoma.

The incidence of *EGFR* 19 Del mutations, 20 ins mutations, and 21 L858R mutations was higher in women than men (28.69% *vs.* 14.63, *P< 0.001*; 3.06% *vs.* 0.61%*, P = 0.005*; 33.15% *vs.* 14.02%, *P< 0.001*; respectively) and higher in never smokers than former/current smokers (25.89% *vs.* 12.75%, *P< 0.001*; 2.37% *vs.* 0.58%, *P = 0.044*; 27.87% *vs.* 13.62%, *P< 0.001*, respectively). *EGFR* 18 G719X mutations were more frequent in older patients than in younger patients (1.63% *vs.* 0.00%, *P = 0.024*), while other *EGFR* mutation subtypes were not significantly associated with age. *EGFR* 20 ins mutations were more common in patients with normal serum tumor markers (3.83% *vs.* 1.05%, *P = 0.037*), and *EGFR* 21 L858R mutations were more frequent in patients with early stage disease (28.19% *vs.* 18.09%, *P< 0.001*). The *EGFR* 19 Del mutation rate was lower in patients with longer tumor long diameters than patients with shorter tumor long diameters (*P = 0.009*). The frequency rate of *EGFR* 19 Del mutations was lower in patients with solid nodules than in patients with partial solid nodules (18.84% *vs.* 37.29%, *P = 0.002*), while the mutation rate of 21 L858R was higher in patients with solid nodules than in patients with partial solid nodules or ground-glass nodules (45.11% *vs.* 33.90% and 45.11% *vs.* 38.46%, *P< 0.001*). Moreover, the frequency of *EGFR* 19-Del mutations (22.54%) and *EGFR* 21 L858R mutations (24.25%) in patients with adenocarcinoma was higher than in patients with other types of NSCLC (all *P< 0.001*) (Table 5).

Multivariate logistic regression analysis was performed on the gender, age, smoking, and drinking history, histopathologic types, serum tumor markers, tumor long diameter, nodule types, and TNM stage of NSLCL patients, and the results showed that *EGFR* mutations were correlated with gender (odds ratio [OR] = 0.359, 95% confidence interval [CI]: 0.237–0.543; *P< 0.001*), smoking history (OR = 1.714, 95% CI: 1.126–2.607; *P = 0.012*), histopathological type (OR = 0.064, 95% CI: 0.015–0.274; *P< 0.001*), and nodule types (OR = 1.974, 95% CI: 1.096–3.556, *P = 0.023*). Meanwhile, *EGFR* mutations were not correlated with age, drinking history, serum tumor markers, tumor long diameter, or TNM stage (*P> 0.05* for all).

**FIGURE 2:**
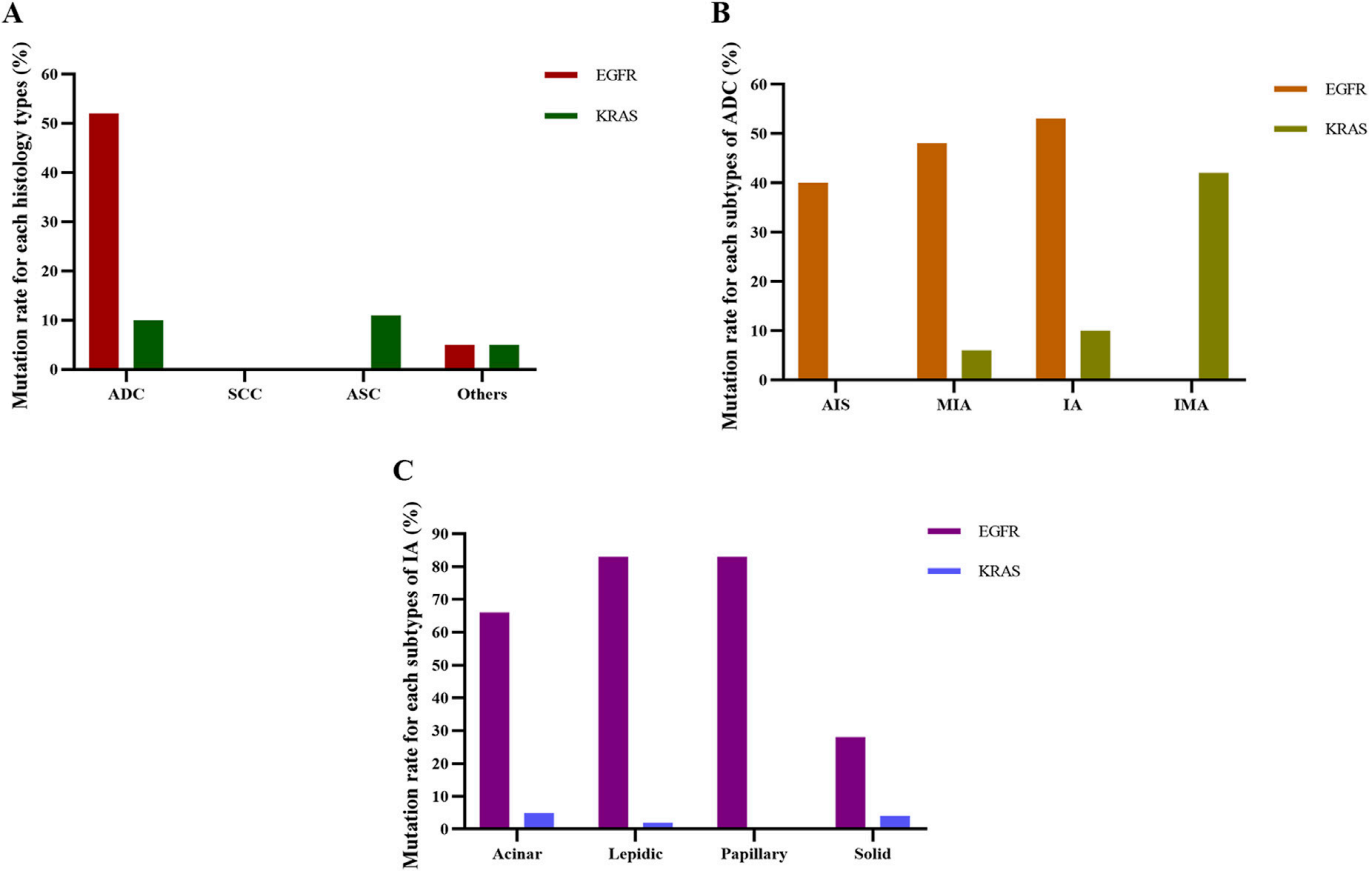
Detection of *EGFR* and *KRAS* mutation rates for each histological subtype. **(A)** Mutation rate for each histology type. **(B)** Mutation rate for each subtype of ADC. **(C)** Mutation rate for each subtype of IA. ADC, adenocarcinoma; SCC, squamous cell carcinoma; ASC, adenosquamous carcinoma; AIS, adenocarcinoma *in situ*; MIA, micro-invasive adenocarcinoma; IA, invasive non-mucinous adenocarcinoma; IMA, invasive mucinous adenocarcinoma.

Next, we aimed to investigate associations between *EGFR* mutation status and the histopathology classification. In the histology types, *EGFR* mutations were most prevalent in ADC (52.79%), while there were no *EGFR* mutations in patients with SCC and ASC (Figure 2A). Among the 771 cases of ADC, the *EGFR* mutation rates of AIS, IMA, IA, and IMA were 40.00%, 48.49%, 53.50%, and 0.00%, respectively (Figure 2B). Of those 249 IA patients, *EGFR* mutations were most common in papillary-predominant (83.87%) and lepidic-predominant adenocarcinoma (83.78%), followed by acinar-predominant (66.88%) and solid-predominant carcinoma (0.00%) (Figure 2C).

Three models of logistic regression were used (as shown in statistical analysis) to further analyze the relationship between histopathology and *EGFR* mutations. The results are shown in Table 6. ADC was associated with an increased probability of *EGFR* mutations in Model 1, Model 2, and Model 3, and the ORs were 43.607, 36.824, and 32.456, respectively (*P< 0.001* for all). Among subtypes of ADC, the frequency of *EGFR* mutations was positively correlated with IA and negatively correlated with MIA in Model 2 and Model 3. Meanwhile, in Model 1, there was no correlation between *EGFR* and IA or MIA. In the IA subtypes, *EGFR* mutations were negatively associated with solid-predominant adenocarcinoma in Model 1, Model 2, and Model 3 (*P< 0.001, P< 0.001, P = 0.004,* respectively) and positively associated with lepidic-predominant adenocarcinoma in Model 1 and Model 2 (*P = 0.033, P = 0.023,* respectively). However, in Model 3, *EGFR* mutations were not significantly associated with lepidic-predominant adenocarcinoma. According to the results of logistic regression analysis, *EGFR* mutations were positively associated with women, non-smokers, ADC, and IA and negatively correlated with solid nodule, MIA, and solid-predominant adenocarcinoma after adjusting for gender, age, smoking history, drinking status, serum tumor markers, pulmonary nodule types, tumor long diameter, and TNM stage.

**TABLE 6:** Univariate and multivariate regression analysis for the correlation of *EGFR* and *KRAS* mutations with histopathologic subtypes of NSCLC.

Histopathology	*EGFR* mutation *P*-value				*KRAS* mutation *P*-value			
	Mutation (%)	Model 1	Model 2	Model 3	Mutation (%)	Model 1	Model 2	Model 3
Histology types								
ADC	407 (52.79)	< 0.001*	< 0.001*	< 0.001*	78 (10.12)	*0.065*	0.009*	0.007*
SCC	0 (0)	*0.998*	*0.998*	*0.998*	0 (0)	*0.057*	*0.998*	*0.998*
ASC	0 (0)	*0.999*	*0.999*	*0.999*	1 (11.11)	*0.870*	*0.847*	*0.872*
Others	2 (5.26)	< 0.001*	< 0.001*	< 0.001*	2 (5.26)	*0.360*	*0.108*	*0.127*
Subtypes of ADC								
AIS	2 (40)	*0.122*	*0.286*	*0.118*	*0 (0)*	*0.452*	*0.507*	*0.568*
MIA	22 (48.89)	*0.589*	0.048*	< 0.001*	3 (6.67)	*0.193*	*0.224*	0.226
IA	382 (53.50)	*0.093*	0.002*	< 0.001*	72 (10.08)	*0.915*	*0.179*	*0.123*
IMA	0 (0)	*0.999*	*0.999*	*0.999*	3 (42.86)	*0.193*	*0.224*	*0.226*
Subtypes of IA								
Acinar	107 (66.88)	*0.525*	*0.689*	*0.936*	9 (5.63)	*0.214*	*0.340*	*0.461*
Lepidic	31 (83.78)	0.033*	0.023*	*0.153*	1 (2.70)	*0.582*	*0.468*	*0.690*
Papillary	26 (83.87)	*0.053*	*0.113*	*0.100*	0 (0)	*0.201*	*0.317*	*0.314*
Solid	6 (28.57)	< 0.001*	< 0.001*	0.004*	1 (4.76)	*0.936*	*0.710*	*0.773*

*indicates *P*-value < 0.05; NSCLC, non-small cell lung cancer; ADC, adenocarcinoma; SCC, squamous-cell carcinoma; ASC, adenosquamous carcinoma; AIS, adenocarcinoma *in situ*; MIA, micro-invasive adenocarcinoma; IA, invasive non-mucinous adenocarcinoma; IMA, invasive mucinous adenocarcinoma.

### 3.4 Relationship between *KRAS* mutations and patient clinicopathologic characteristics

Among 81 patients with *KRAS* mutations, the distribution of the mutations was 66 cases in *KRAS* exon 2 G12C, G12R, G12V, G12A, G13C (81.48%) and 15 cases in *KRAS* exon 2 G12S, G12D (18.52%). As Table 7 shows, the mutation rate of *KRAS* was higher in men than women (16.06% *vs.* 0.56%, *P< 0.001*), higher in older patients than younger patients (11.41% *vs.* 6.94%, *P = 0.028*), higher in current or former smokers than non-smokers (16.81% *vs.* 4.55%, *P< 0.001*), higher in abnormal serum tumor markers than normal serum tumor markers (10.78% *vs.* 4.92%, *P = 0.017*), and higher in advanced stage than early stage (12.45% *vs.* 5.04%, *P< 0.001*). *KRAS* mutations were more common in patients with longer tumor long diameters than in patients with shorter tumor long diameters (*P = 0.006*). No significant correlation was observed in total *KRAS* mutations with drinking habits and histology types.

**TABLE 7:** Relationship between *KRAS* gene mutations and clinicopathologic characteristics in patients with NSCLC.

Clinicopathologic characteristic	No. of patients			
	No. of patients	Total *KRAS* mutations	G12C, G12D, G12V, G12A, and G13C	G12S, G12D
No. of patients		81	66	15
Sex (*P*)		< 0.001*	< 0.001*	< 0.001*
Male	492	79	64	15
Female	359	2	2	0
Age (P)		0.028*	*0.125*	*0.078*
≤ 60	360	25	22	3
> 60	491	56	44	12
Smoking habit (*P*)		< 0.001*	< 0.001*	*0.121*
Never	506	23	17	6
Former/current	345	58	49	9
Drinking habit (*P*)		*0.066*	*0.059*	*0.680*
Never	756	67	54	13
Former/current	95	14	12	2
Serum tumor markers (*P*)		0.017*	*0.053*	*0.214*
Normal	183	9	8	1
Abnormal	668	72	58	14
Pulmonary nodule types (*P*)		0.035*	*0.150*	*0.641*
Solid nodule	727	77	62	15
Part-solid nodule	59	2	2	0
Ground-glass nodule	65	2	2	0
Tumor long diameter (*P*)		0.006*	*0.051*	0.01*
With alteration (mean ± SD)		4.38 ± 2.94	4.21 ± 3.01	5.13 ± 2.61
Without alteration (mean ± SD)		3.42 ± 2.35	3.46 ± 2.37	3.49 ± 2.42
Histology types (*P*)		*0.168*	*0.266*	*0.581*
ADC	771	78	63	15
SCC	33	0	0	0
ASC	9	1	1	0
Others	38	2	2	0
TNM stage (*P*)		< 0.001*	0.009*	0.009*
0 + I + II	337	17	16	1
III + IV	514	64	50	14

*indicates *P*-value < 0.05; NSCLC, non-small cell lung cancer; ADC, adenocarcinoma; SCC, squamous cell carcinoma; ASC, adenosquamous carcinoma.

Both *KRAS* exon 2 G12C, G12R, G12V, G12A, G13C mutations and G12S, G12D mutations were more common in men than women (13.01% *vs.* 0.56%, 3.05% *vs.* 0.00%, both *P< 0.001*), and more in advanced stage than early stage (9.73% *vs.* 4.75%, 2.72% *vs.* 0.30%, both *P = 0.009*). Current or former smokers had a higher *KRAS* 2 G12C, G12V, G12V, G12A, G12A, G13C mutation rate than non-smokers (14.20% *vs.* 3.36%, *P< 0.001*). However, there was no significant association between G12S, G12D mutations and smoking history. In addition, *KRAS* exon 2 G12S, G12D mutations were significantly associated with longer tumor long diameter (*P = 0.01*), while there was no correlation between G12C, G12R, G12V, G12A, G13C mutations and tumor long diameter. No significant associations were observed between *KRAS* exon 2 G12C, G12R, G12V, G12A, G13C mutations and G12S, G12D mutations and age, drinking habit, serum tumor markers, pulmonary nodule types, histology types, or subtypes of invasive adenocarcinoma (Table 7).

Multivariate logistic regression analysis was performed on the gender, age, smoking, and drinking history, histopathologic types, serum tumor markers, tumor long diameter, nodule types, and TNM stage of NSLCL patients, and the results showed that *KRAS* mutations were correlated with gender (OR = 0.029, 95% CI: 0.007–0.117; *P< 0.001*) and tumor long diameter (OR = 1.120, 95% CI: 1.025–1.223; *P = 0.012*). Meanwhile, *KRAS* mutations were not correlated with age, smoking status, drinking history, serum tumor markers, histological types, nodule types, or TNM stage.

In the histology types, *KRAS* mutations were most common in ASC (11.11%), followed by ADC (10.12%), other types (5.26%), and SCC (0.00%) (Figure 2A). Among ADC patients, the frequency of *KRAS* mutation in AIS, IMA, IA, and IMA was 0.00%, 6.67%, 10.08%, and 42.86%, respectively (Figure 2B). Of the IA patients, *KRAS* mutations were most frequent in acinar-predominant (5.63%), followed by solid-predominant adenocarcinoma (4.76%), lepidic-predominant (2.70%), and papillary-predominant carcinoma (0.00%) (Figure 2C). As with the *EGFR* analysis, we used Model 1, Model 2, and Model 3 to further analyze the relationship between histopathology and *KRAS* mutations. The results showed that ADC was significantly associated with a reduced probability of *KRAS* mutations in Model 2 and Model 3 (*P = 0.009 and P = 0.007*, respectively) (Table 6). According to the results of logistic regression analysis, *KRAS* mutations were positively associated with men and larger tumor long diameter and negatively associated with lung adenocarcinoma after adjusting for gender, age, smoking history, drinking status, serum tumor markers, pulmonary nodule types, tumor long diameter, and TNM stage.

### 3.5 Status of *MET*, *ALK*, *ROS1*, *HER2*, *RET*, *BRAF*, *NRAS*, and *PIK3CA* alterations and associations with clinicopathologic characteristics

As shown in Table 8, the frequency of *ALK* rearrangements, *HER2* mutations, and *RET* fusions was significantly higher in women than in men (8.08% *vs.* 3.46%, *P = 0.003*; 4.18% *vs.* 1.02%, *P = 0.003*; 3.34% *vs.* 1.22%, *P = 0.034*; respectively). In older patients, there was a significantly higher mutation rate of *MET* exon 14 skipping mutations (3.67% *vs.* 0.28%, *P< 0.001*) and a lower frequency of *ALK* rearrangements and *HER2* mutations (2.85% *vs.* 8.89%, *P< 0.001*; 1.22% *vs.* 3.89%, *P = 0.011*; respectively). *ALK* rearrangements and *HER2* mutations were more common in non-smokers than current or former smokers (7.31% *vs.* 2.61%, *P = 0.003*; 3.56% *vs.* 0.58%, *P = 0.005;* respectively). *ALK* rearrangements were more frequent in non-drinkers (5.95% *vs.* 1.05%, *P = 0.047*) and patients with advanced stages (6.81% *vs.* 3.26%, *P = 0.025*), and *RET* fusions were more common in patients with normal tumor markers (4.92% *vs.* 1.35%, *P = 0.007*). *HER2* mutations were significantly positively associated with shorter tumor long diameters (*P = 0.034*). *MET* exon 14 skipping mutations were more common in patients with ASC (1/33, *P = 0.018*), while *PIK3CA* mutations were more frequent in patients with SCC (1/33, *P = 0.049*). No significant associations of these eight target gene alterations were observed with subtypes of invasive adenocarcinoma or nodule types, other than the mutation rate of *ALK* fusions was higher in solid-predominant adenocarcinoma patients than that of acinar or lepidic-predominant adenocarcinoma patients (23.81% *vs.* 3.13%, 23.81% *vs.* 0.00%, *P = 0.002*).

**TABLE 8:** Relationship between eight other targeted gene alterations and clinicopathologic characteristics in patients with NSCLC.

Clinicopathologic characteristic	No. of patients								
	No.	RET	ALK	ROS1	HER2	MET	BRAF	NRAS	PIK3CA
No. of patients		18	46	16	20	19	4	2	1
Sex (*P*)		0.034*	0.003*	*0.250*	0.003*	*0.059*	*0.642*	*0.512*	*0.393*
Male	492	6	17	7	5	15	3	2	1
Female	359	12	29	9	15	4	1	0	0
Age (*P*)		*0.767*	<0.001*	*0.254*	0.011*	<0.001*	*0.642*	*0.825*	*0.423*
≤ 60	360	7	32	9	14	1	1	1	1
> 60	491	11	14	7	6	18	3	1	0
Smoking habit (*P*)		*0.110*	0.003*	*0.201*	0.005*	*0.888*	*0.309*	*0.785*	*0.405*
Never	506	14	37	12	18	11	1	1	0
Former/current	345	4	9	4	2	8	3	1	1
Drinking habit (*P*)		*0.599*	0.047*	*0.303*	*0.599*	*0.929*	*0.064*	*0.616*	*0.112*
Never	756	17	45	16	19	17	2	2	0
Former/current	95	1	1	0	1	2	2	0	1
Tumor markers (*P*)		0.007*	*0.485*	*0.206*	*0.078*	*0.741*	*0.583*	*0.459*	*0.600*
Normal	183	9	8	6	8	3	0	0	0
Abnormal	668	9	38	10	12	16	4	2	1
Nodule types (*P*)		*0.151*	*0.139*	*0.390*	*0.200*	*0.903*	*0.710*	*0.843*	*0.918*
Solid nodule	727	15	43	14	15	17	4	2	1
Part-solid nodule	59	3	0	0	2	1	0	0	0
Ground-glass nodule	65	0	3	2	3	1	0	0	0
Tumor long diameter (*P*) (mean ± SD)		*0.761*	*0.761*	*0.972*	0.034*	*0.704*	*0.704*	*0.393*	*—*
With alterations		3.69 ± 2.43	3.67 ± 2.73	3.54 ± 2.52	2.38 ± 1.57	3.73 ± 2.15	4.58 ± 2.06	2.05 ± 1.34	7.50
Without alterations		3.51 ± 2.43	3.51 ± 2.41	3.52 ± 2.43	3.54 ± 2.44	3.51 ± 2.44	3.51 ± 2.43	3.52 ± 2.43	3.51 ± 2.43
Histology types (*P*)		*0.441*	*0.656*	*0.588*	*0.863*	0.018*	*0.937*	*0.976*	0.049*
ADC	771	16	44	16	20	15	4	2	0
SCC	33	0	0	0	0	1	0	0	1
ASC	9	0	0	0	0	2	0	0	0
Others	38	2	2	0	0	1	0	0	0
Subtypes of IA (*P*)		*0.520*	0.002*	*0.103*	*0.772*	*0.832*	*—*	*0.906*	*—*
Acinar	160	4	5	2	2	3	0	1	0
Lepidic	37	0	0	1	0	1	0	0	0
Papillary	31	0	1	0	0	0	0	0	0
Solid	21	0	5	2	0	0	0	0	0
TNM stage (*P*)		*0.950*	0.025*	*0.391*	*0.617*	*0.240*	*0.157*	*0.763*	*0.418*
0 + I + II	337	7	11	8	9	10	0	1	0
III + IV	514	11	35	8	11	9	4	1	1

*indicates *P*-value < 0.05; NSCLC, non-small cell lung cancer; ADC, adenocarcinoma; SCC, squamous cell carcinoma; ASC, adenosquamous carcinoma.

## 4 Discussion

In this study, we assayed and investigated the status of *EGFR*, *KRAS*, *MET*, *ALK*, *ROS1*, *HER2*, *RET*, *BRAF*, *NRAS*, and *PIK3CA* somatic driver gene alterations derived from 851 Chinese NSCLC patients. The intent was to analyze the 10 targeted gene alterations status and their correlation with clinicopathological characteristics of NSCLC patients. We found that most NSCLC patients have at least one gene mutation, with the first two highest aberrant target oncogenes being *EGFR* (48.06%) and *KRAS* (9.51%). In patients with adenocarcinoma, the *EGFR* and *KRAS* mutation rates were 52.79% and 10.12%, respectively. Among women, 69.36% had *EGFR* mutations, and only 0.56% had *KRAS* mutations. Meanwhile, 32.52% of men had *EGFR* mutations, and 16.06% had *KRAS* mutations. *EGFR* and *KRAS* mutations were present in 61.26% and 4.55% of non-smokers, while in former/current smokers, the mutation rates were 28.70% and 16.81%, respectively. Furthermore, through three logistics regression models, we found that the probability of *EGFR* mutations was positively correlated with women, non-smokers, lung adenocarcinoma, and IA subtype of adenocarcinoma, while *EGFR* mutations were negatively correlated with solid nodule, the MIA subtype of adenocarcinoma, and the solid-predominant adenocarcinoma subtype of IA. *KRAS* mutations were positively associated with men and longer tumor long diameter and negatively associated with lung adenocarcinoma.

Some previous studies have analyzed the association of driver gene alterations with clinicopathologic features in patients with NSCLC (Chen et al., 2022). The most common mutation in NSCLC is an *EGFR* mutation, and there are significant regional differences in *EGFR* mutations in lung cancer patients. The prevalence of *EGFR* mutations in Asian and European NSCLC patients was 49.1% and 12.8%, respectively (Melosky et al., 2022). The most frequently aberrant target oncogene in our study was *EGFR* (48.06%), which was similar to that in East Asian patients (Liu et al., 2017; Melosky et al., 2022). However, it was lower than another study from China, which had an *EGFR* mutation rate of 57.7% (Li et al., 2020). This may be because the patients enrolled by Li et al. were mainly in the early stage, with TNM stage I and II patients accounting for 64.3%, while only 39.6% of the patients in our study were in the early stages of the disease. In this study, overall *EGFR* mutations and subtypes other than exon 18 G719 X and exon 21 L861Q mutations were more frequent in women, non-smokers, and patients with adenocarcinoma, which is consistent with previous findings (Melosky et al., 2022; Li et al., 2020; Gao et al., 2020). Some studies found no association between smoking status and *EGFR* mutations (Liu et al., 2017; Ess et al., 2017). This reminds us that the smoking index should be recorded clearly in future studies. Like most studies, we found that *EGFR* mutation and its subtypes were not associated with age, except for subtype exon 18 G719X (Liu and Xiong, 2021). This may be due to the absence of patients younger than 60 years in the eight patients with exon 18 G719X mutation. Similar to the results of Hong et al. (2016), we also found a lower mutation rate of *EGFR* in solid nodules than in part-solid nodules or ground-glass nodules, while the result of Zhao et al. was contrary (Zhao et al., 2018). This may be because Zhao classifies CT nodule types into two categories: ground-glass opacity (GGO)/ground-glass nodule (GGN) and no GGO/GGN, while we classified them into solid nodule, partial solid nodule, and ground-glass nodule.

Many studies have indicated that the *EGFR* mutation rate was higher in lung adenocarcinoma patients, but different studies had different methods for further pathologic classification of adenocarcinoma (Melosky et al., 2022). In this study, we used three logistic regression models to explore the association between histopathologic typing and NSCLC driver genes according to the latest WHO histologic classification (5th). In all three logistic regression models, we found *EGFR* was positively associated with ADC and subtype IA in ADC and negatively correlated with subtype MIA in ADC and subtype solid-predominant in IA. Among subtypes of IA, *EGFR* mutations were more common in the papillary, lepidic, and papillary-predominant subtypes, although no statistical association was found in the full adjusted logistic regression model.

The point mutations of *EGFR* exon 20 mainly include T790M and S768I, which are insensitive mutations (Romero, 2023; Wu et al., 2017). Previous studies have reported that most patients using EGFR-TKIs will develop resistance 9–14 months after treatment, with *EGFR* exon 20 T790M mutation being the most common (Rosell et al., 2012; Planchard et al., 2023). This also explains why the six patients with the T790M mutation in this study all had the *EGFR*-sensitive mutations, namely 19-Del (5/6) or L868R (1/6), and had a history of targeted therapy for EGFR-TKIs. The mutation rate of S768I accounts for about 1%–2% of *EGFR* mutations, including single mutations and combinations with other mutations (Kobayashi and Mitsudomi, 2016). In this study, nine of 851 patients developed the *EGFR* exon 20 S768I mutation (1.05%), all in combination with G719X, 19-Del, or L861R mutations. Studies have shown that when S768I was combined with the sensitive mutation 19-Del or L858R, the response effect of the sensitive mutation was not affected (Hao et al., 2023; Lin et al., 2023). Meanwhile, patients with S768I combined with the insensitive G719X mutation showed an enhanced response to EGFR-TKIs (Morimoto et al., 2023; Svaton et al., 2015). However, these are all small cohorts or even case reports, and studies with larger samples are needed to further explore the mechanism and clarify the therapeutic effect of S768I combined with other mutations.

Similar to the results of the current study, the *KRAS* mutation rate was higher in men, older patients, and current or former smokers by a chi-square test (Xu et al., 2011; Zhao et al., 2018). However, these studies did not conduct further univariate or multivariate regression analysis of the correlation. In this study, regression analysis showed that *KRAS* mutations had no statistical correlation with age and smoking status. Moreover, in the chi-square test and univariate regression analysis, there was no correlation between *KRAS* and histopathologic type. After preliminary and full adjustment, *KRAS* mutations were negatively correlated with the ADC subtype.

In this study, we found that *ALK*, *RET*, and *ROS1* fusions accounted for 5.41%, 2.12%, and 1.88% of the total driver gene alterations, respectively. Their fusion rate in lung adenocarcinoma patients was 5.71%, 2.08%, and 2.59%, respectively, which was similar to previous studies (Gou and Wu, 2014; Li et al., 2021). *ALK* translocations were significantly more common in women, younger patients, non-smokers, non-drinkers, and patients in the advanced stages. Moreover, in the subtypes of invasive non-mucinous adenocarcinoma, the frequency of *ALK* translocations was significantly higher in patients with solid-predominant adenocarcinoma than in the other three subtypes of IA. *RET* fusions were significantly more frequent in women and patients with normal serum tumor markers. We found no significant relationship between clinicopathological features and *ROS1* fusions. *HER-2* mutations were more common in women, younger patients, non-smokers, and patients with a smaller tumor long diameter. *MET* exon 14 skipping mutations were more frequent in older patients and ASC.

There are some limitations to our study. First, this study is a single-center retrospective study, which can prove correlation but not causation. Future prospective cohort studies are needed to explore causal associations. Second, due to the brevity of the follow-up time, patient prognosis was not tracked in this study. Future studies are needed to analyze the relationship between gene alterations and patient clinical outcomes.

## 5 Conclusion

Overall, our work demonstrated that the probability of *EGFR* mutations was positively correlated with women, non-smokers, lung adenocarcinoma, and the IA subtype of adenocarcinoma, while *EGFR* mutations were negatively correlated with solid nodule, the MIA subtype of adenocarcinoma, and the solid-predominant adenocarcinoma subtype of IA. *KRAS* mutations were positively associated with men and longer tumor long diameter and negatively associated with lung adenocarcinoma. Specifically, our work may help thoracic surgeons, respirologists, and oncologists use the information obtained in this study to guide targeted treatments.

## Data Availability

The original contributions presented in the study are included in the article/Supplementary Material; further inquiries can be directed to the corresponding author.

## Supplementary material

SUPPLEMENTARY FIGURE S1 Distribution and proportion of gene alterations in 851 patients. (**A)** Distribution of gene alterations in 851 patients with NSCLC. **(B)** Proportions of 10 target gene alterations in 851 patients with NSCLC. NSCLC, non-small cell lung cancer.
