# Correction: Driver gene alterations in NSCLC patients in southern China and their correlation with clinicopathologic characteristics

**DOI:** 10.3389/fgene.2026.1794164

**Published:** 2026-01-30

**Authors:** Lingna Deng, Jinbang Li, Zhanlong Qiu, Yanfen Wang

**Affiliations:** Department of Pathology, The Affiliated Qingyuan Hospital (Qingyuan People’s Hospital), Guangzhou Medical University, Qingyuan, China

**Keywords:** EGFR, genomic alterations, non-small cell lung cancer, targeted therapy, KRAS

The funder “Medical Science and Technology Research Foundation of Guangdong Province (grant number A2023166)” was erroneously omitted.

The correct **funding** statement reads:

The author(s) declared that financial support was received for this work and/or its publication. The research was supported by the Medical Science and Technology Research Foundation of Guangdong Province (grant number A2023166) and the Affiliated Qingyuan Hospital (Qingyuan People’s Hospital), Guangzhou Medical University (grant number 2022-11217).

The original article has been updated.

