# Supplementary material for: Driver gene alterations in NSCLC patients in southern China and their correlation with clinicopathologic characteristics
Source: Front Genet. 2024 Sep 19;15:1455502. doi: 10.3389/fgene.2024.1455502 (PMC11446855; doi:10.3389/fgene.2024.1455502)
Supplement: Supplementary file 1 [file Table1.docx]

**Driver genes alterations in NSCLC patients in** **southern China and** **there correlation with clinicopathologic characteristics**

**Lingna Deng^1^, Jinbang Li^1^, Zhanlong Qiu^1^, Yanfen Wang^1*^**

**^1^** Department of Pathology, The Affiliated Qingyuan Hospital (Qingyuan People’s Hospital), Guangzhou Medical University. No.35, Yinquan North Road, Qingcheng District, Qingyuan Guangdong, China, 511518

**^*^ Corresponding Author:** Yanfen Wang, E-mail: 2022695043@gzhmu.edu.cn

**Table of the supporting information**

**Table S1** Clinicopathological features of NSCLC patients with two co-existing *EGFR* mutation sites

| Case | Mutation sites | Age/Gender | Smoking | Histology type | Subtypes of ADC | Subtypes of IA | History of EGFR-TKI targeted therapy |
| --- | --- | --- | --- | --- | --- | --- | --- |
| 1 | S768I/G719X | 55/M | Never | ADC | IA | Acinar | Never |
| 2 | S768I/G719X | 61/M | Never | ADC | IA | / | Never |
| 3 | S768I/G719X | 62/M | Current | ADC | IA | / | Never |
| 4 | S768I/G719X | 50/M | Never | ADC | MIA | / | Never |
| 5 | S768I/G719X | 59/FM | Never | ADC | IA | / | Never |
| 6 | S768I/G719X | 67/M | Current | ADC | IA | / | Never |
| 7 | S768I/L858R | 61/M | Current | ADC | IA | Acinar | Never |
| 8 | S768I/L858R | 61/M | Never | ADC | IA | Acinar | Never |
| 9 | S768I/19-Del | 80/M | Never | ADC | IA | Acinar | Never |
| 10 | T790M/19-Del | 72/M | Never | ADC | IA | / | Gefitinib |
| 11 | T790M/19-Del | 74/FM | Never | ADC | IA | / | Gefitinib |
| 12 | T790M/19-Del | 70/FM | Never | ADC | IA | / | Gefitinib |
| 13 | T790M/19-Del | 69/FM | Never | ADC | IA | / | Gefitinib |
| 14 | T790M/19-Del | 52/M | Former | ADC | IA | / | Osimertinib |
| 15 | T790M/L858R | 67/FM | Never | ADC | IA | Acinar | Gefitinib |
| 16 | 19-Del/L858R | 62/FM | Never | ADC | IA | / | Never |
| 17 | 19-Del/L858R | 64/M | Never | ADC | IA | Acinar | Never |
| 18 | G719X /L861Q | 47/M | Never | ADC | IA | Acinar | Never |

NSCLC, non-small cell lung cancer; ADC, adenocarcinoma; MIA, micro invasive adenocarcinoma; IA, invasive non-mucinous adenocarcinoma.
