# Supplementary figures and images for: Driver gene alterations in NSCLC patients in southern China and their correlation with clinicopathologic characteristics
Source: Front Genet. 2024 Sep 19;15:1455502. doi: 10.3389/fgene.2024.1455502 (PMC11446855; doi:10.3389/fgene.2024.1455502)

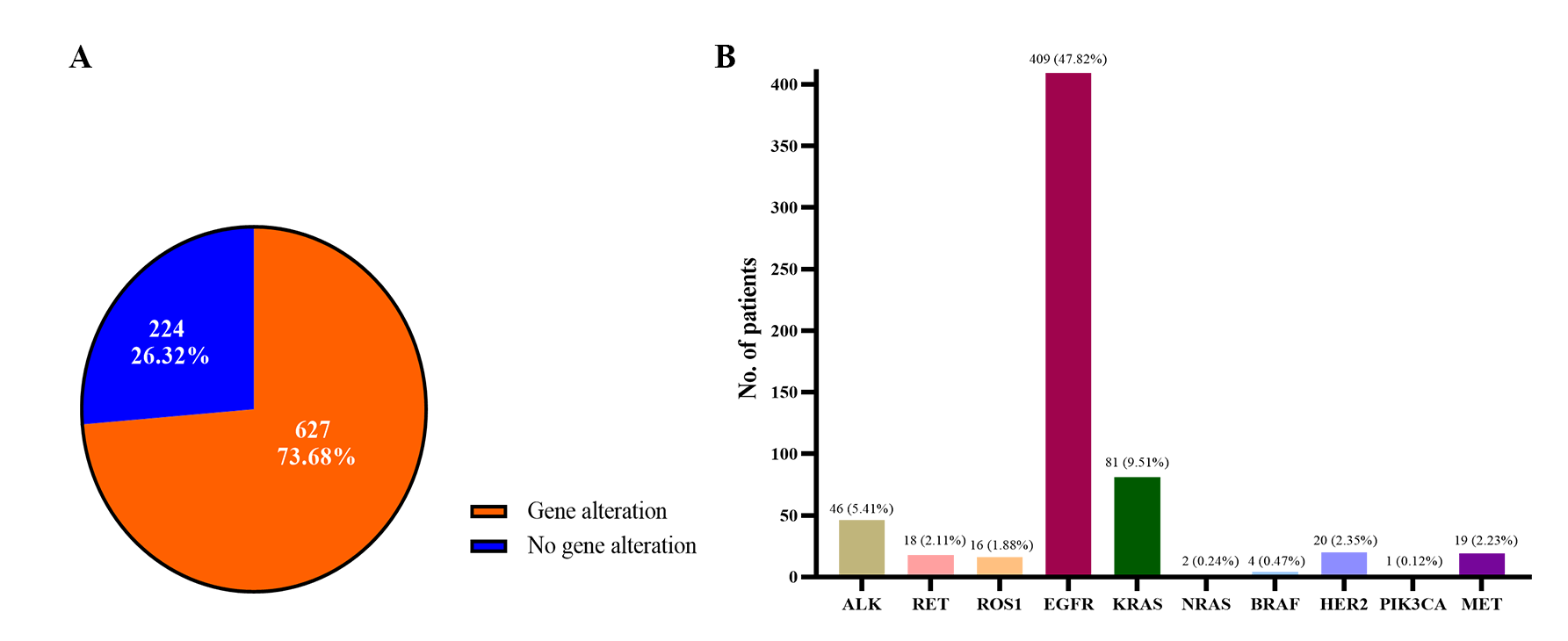

Supplement: Supplementary file 2 [file Image1.TIF]
